# Prevalence, Pattern and Genetic Diversity of Rotaviruses among Children under 5 Years of Age with Acute Gastroenteritis in South Africa: A Systematic Review and Meta-Analysis

**DOI:** 10.3390/v13101905

**Published:** 2021-09-23

**Authors:** Cornelius A. Omatola, Ropo E. Ogunsakin, Ademola O. Olaniran

**Affiliations:** 1 Discipline of Microbiology, School of Life Sciences, College of Agriculture, Engineering and Science, University of KwaZulu-Natal (Westville Campus), Private Bag X54001, Durban 4000, South Africa; omatola.ca@ksu.edu.ng; 2 Discipline of Public Health Medicine, School of Nursing and Public Health, College of Health Sciences, University of KwaZulu-Natal (Westville Campus), Private Bag X54001, Durban 4000, South Africa; ogunsakinR@ukzn.ac.za

**Keywords:** rotavirus, diarrhea, rotavirus vaccine, disease burden, genotype diversity, meta-analysis, South Africa

## Abstract

Rotavirus is the most significant cause of severe acute gastroenteritis among children under 5 years of age, worldwide. Sub-Saharan Africa particularly bears the brunt of the diarrheal deaths. A meta-analysis was conducted on 43 eligible studies published between 1982 and 2020 to estimate the pooled prevalence of rotavirus infection and changes in the main rotavirus strains circulating before and after vaccine introduction among under-five children in South Africa. The pooled national prevalence of rotavirus infection was estimated at 24% (95% CI: 21–27%) for the pre-vaccination period and decreased to 23% (95% CI: 21–25%) in the post-vaccination period. However, an increased number of cases was observed in the KwaZulu-Natal (21–28%) and Western Cape (18–24%) regions post-vaccination. The most dominant genotype combinations in the pre-vaccine era was G1P[8], followed by G2P[4], G3P[8], and G1P[6]. After vaccine introduction, a greater genotype diversity was observed, with G9P[8] emerging as the predominant genotype combination, followed by G2P[4], G12P[8], and G1P[8]. The introduction of the rotavirus vaccine was associated with a reduction in the burden of rotavirus-associated diarrhea in South Africa, although not without regional fluctuation. The observed changing patterns of genotype distribution highlights the need for ongoing surveillance to monitor the disease trend and to identify any potential effects associated with the dynamics of genotype changes on vaccine pressure/failure.

## 1. Introduction

Rotavirus is the leading etiology of severe acute gastroenteritis accounting for approximately 258 million morbidity cases and 128,000 diarrheal deaths annually among neonates and children younger than 5 years, in both developed and developing countries [1]. Sub-Saharan African countries bear the highest rotavirus associated diarrheic burden, as they carry >80% of the global rotavirus mortality [1,2]. After respiratory tract infections, diarrheal disease is ranked the second most frequent cause of childhood mortality across the globe [3]. Of all diarrheic agents, rotavirus is recognized as the most significant causal agent of severe gastroenteritis in young children worldwide [4,5]. In several countries where RV vaccination was introduced, noroviruses (NoV) have become the most important cause of viral acute gastroenteritis (AGE) [6,7,8]. In South Africa, diarrheal diseases are currently rated as the third major cause of death in children under 5 years [9], and before the use of rotavirus vaccine, most children in the country became infected with rotavirus before their third birthday [10,11]. Rotaviruses exhibit a high level of diversity in terms of genotypes circulating across the globe. Every year, new strains emerge because of high frequencies of genetic changes that accompany genomic re-assortment, gene recombination, accumulation of point mutations, and interspecies transmission mechanism events [12,13]. Nine genetic groups (A–J) of rotaviruses have been differentiated, with new species K and L being proposed [14]. Rotaviruses of all groups infect animals, while only those of groups A–C infect humans [15]. The genetic differences of the two outer capsid proteins, VP7 and VP4, are used to classify the virus into G (for glycoprotein) and P (for protease-sensitive) genotypes, respectively [16]. To date, 36 different G genotypes and 51 different P genotypes have been described by RT-PCR and sequencing techniques [17]. Six G/P combinations: G1P[8], G2P[4], G3P[8], G4P[8], G9P[8], and G12P[8] or G9P[8] are the most prevalent combinations detected in humans globally [18,19,20,21,22,23,24]. Previously, uncommon rotavirus genotypes such as G1P[4], G2P[8], G9P[4], G12P[4], G8P[6], G8P[8], and G12P[6] have in recent times acquired epidemiological relevance on the African continent [13,25] and increased strain surveillance is needed to monitor the prevalence and potential changes of the dominant G and P types circulating in a given region.

Rotavirus is excreted in high amounts in stool and transmitted mainly by the fecal–oral route person-to-person, through contact with contaminated fomites, and consumption of contaminated food or water [26]. The infection is common in settings characterized by poor water, sanitation, and hygiene [26,27]. The current global efforts in ensuring a significant reduction in disease burden in the low-income countries of Africa and Asia with notable high birth cohorts may be difficult due to the limited access to good sanitation, safe water, medical treatment, and current delay in the introduction of the new rotavirus vaccine to national immunization routine [28].

Two orally administered live rotavirus vaccines (Rotarix and RotaTeq) have been licensed for global prevention and control of rotavirus infection among children [26]. The vaccine has been introduced in 37 countries in sub-Saharan Africa [29]. Recently, Rotasiil and Rotavac were also licensed internationally and prequalified by WHO. Both vaccines were introduced by India and African countries [30]. Before global vaccine use, rotavirus infection was implicated in 2.1–3.2 million diarrheal morbidities, with 55,000–70,000 cases necessitating hospitalization each year [31,32]. Epidemiological data in the post-vaccination era have shown a significant decline in the severity of rotavirus gastroenteritis burden and mortality in many countries [26,33]. South Africa included the rotavirus vaccine in the childhood immunization programs in August 2009 [7] and the country is among the nations in sub-Saharan Africa where the early impact of vaccine introduction has been documented [2,34]. Despite the decline in the national rotavirus associated diarrheic burden in the past decade, some recent reports of post-vaccination licensure studies in South Africa have documented alarming rates of rotavirus-associated diarrheal morbidity and hospitalization in some regions of the country, despite improvements in vaccine coverage [11,35]. Generally, studies have shown that the efficacy of the oral vaccine is higher in developed countries than in developing nations due to reduced oral vaccine immunogenicity by prevalent factors such as nutritional deficiencies [36] and fecal contamination leading to poor water, sanitation and hygiene, which substantially contribute to intestinal pathology [37].

The appraisal of the current burden of rotavirus disease among South African children and the impacts of rotavirus vaccination necessitates robust and reliable peer-reviewed epidemiological studies with data on the prevalence, incidence, and molecular types in local distribution. A review of pre- and post-vaccine studies across regions of the country will help to understand the genetic diversity of rotavirus and potential changes in the local epidemiology of rotavirus disease. Such information makes it easier to identify the region of significant burden, and also generate evidence-based information from the pool of studies needed for close monitoring of genetic variants in events of vaccine pressure/failure as well as inform policy decision on possible review of the vaccine in the area.

To date, there have been no meta-analysis studies on the nationally representative prevalence of rotavirus disease and genotype distribution across the nine provinces of South Africa since rotavirus vaccination was introduced into routine immunization in 2009. Previous systematic reviews by Waggie et al. [38] on rotavirus studies from 1996–2006 in Africa reported a rotavirus prevalence of 25% among African children under 5 years. Although this information is important, it cannot be extrapolated to the disease burden in South African children. Therefore, this study reviewed articles published over nearly four decades (1982–2020) to assess the impact of rotavirus vaccine introduction on the national and local epidemiology of rotavirus disease among diarrheic children under 5 years in South Africa.

## 2. Methods

### 2.1. Literature Search Strategy

This review was carried out in line with the established PRISMA (preferred reporting items for systematic reviews and meta-analyses) protocol [39]. A systematic search was carried out in PubMed/Medline, Science direct, Google SCHOLAR, Cochrane Library, EmBase, WHO, Gavi, and Africa journals online (AJOL) databases for articles published from January 1982 to July 2020. Other studies were identified in the National Institute for Communicable Diseases (NICD) website. Publications were identified using the search terms “rotavirus”, “rotavirus vaccine”, “diarrhea”, “prevalence”, “burden”, “genotypes”, “epidemiology”, “South Africa” and related terms. Additionally, references in identified articles were further screened for publications relevant to the study.

### 2.2. Inclusion and Exclusion of Studies

Included studies were full texts reported in English or other relevant languages on rotavirus infection among <5-year-old children in South Africa that satisfied the case definition described by WHO [26]. Briefly, they had to have recruited cases with acute watery diarrhea, defined as three or more loose or watery stools in a 24-h period. Studies are included if they had used one or a combination of enzyme immune assay (EIA) or enzyme-linked immunosorbent assay (ELISA), electron microscopy, or reverse transcription–polymerase chain reaction (RT-PCR) for the identification of rotavirus in samples. In the case of duplicate studies, only the ones with adequate datasets were included. Additionally, data were extracted from the most recent available publication where multiple publications resulted from the same study. To appraise the burden of rotavirus disease, publications with fewer than 30 diarrheic children and in which study duration was less than 6 months were excluded. Also excluded were studies with inadequate datasets and in which patient selection was from studies outside South Africa. Studies on asymptomatic and immune-compromised children, as well as children older than 5 years, were excluded. Reports of rotavirus genotypes characterizations were finally retrieved to assess the distribution of rotavirus strains in circulation.

### 2.3. Data Extraction and Quality Assessment

Data extracted from the retrieved studies were entered into a Microsoft Office Excel database. To avoid being mixed up, each study was assigned a number and the following information were collected: author details, year of publication, province/region of South Africa where the study took place, study setting, study design, duration/period of study, sample size, the definition of diarrheal cases, inclusion criteria, assay method, sampling strategy, age band, study setting (community, outpatient department, or hospital-based), seasonality, number of rotavirus positive cases, typing method (if any), numbers of genotyped samples, and numbers positive for G and P-type combinations. We found that for studies where both ELISA and RT-PCR were available, the later was applied on genotyping ELISA positive samples. The ELISA positive samples were included in computing meta-analysis proportions. All studies included in the description of genotypes employed RT-PCR. To rule out data duplication, studies reported from the same regions or areas were cross-referenced by location. The methodological qualities of each article were assessed based on a 12-point scoring system using the modified checklists of Downs and Black [40]. Another relevant quality assessment tool developed by Downes et al. [41] for assessing the quality of a cross-sectional study was also adapted for the study. The first and second authors (Cornelius Arome Omatola (CAO) and Ropo Ebenezer Ogunsakin (REO)) carried out the assessment independently, and any disagreement was resolved by discussion. Scores were assigned on the basis of the following quality checklists: study objective clearly described, design of study indicated, the representativeness of participants in the population from which they were recruited, participants accrued during the same period, sample size justified, management of missing data, age, gender and other characteristics explored/reported (e.g., were confounding variables reported, were rotavirus detection method reported, were potential biases reported, was outcome clearly described?). The studies were categorized into pre-vaccination and post-vaccination periods. South Africa introduced the rotavirus vaccine in August 2009. Consequently, all studies conducted from 1982 to before August 2009 were considered to be pre-vaccination period studies. Studies conducted from a year after vaccine implementation were considered to be post-vaccination period studies.

### 2.4. Statistical Data Analysis

Data were extracted into a Microsoft Excel spreadsheet, and analysis was carried out using R statistical software. Because substantial heterogeneity was expected, random-effects estimates were employed, since the samples were from general populations [42,43,44]. Heterogeneity among reported prevalence was assessed by computing *p*-values of Higgins’s I^2^ statistics; I^2^ was considered significant at *p*-value < 0.05. The DerSimonian and Laird’s random-effects meta-analysis model was used to determine the pooled effect size since the true effect is not the same in all studies [42]. We dealt with heterogeneity of data using subgroup analysis, meta-regression, and sensitivity analysis. Subgroup analysis was performed based on study settings, province, age, and period. Additionally, to understand the sources of heterogeneity, univariate meta-regression analysis was conducted for sample size, publication year, and study design. A forest plot was used to describe pooled prevalence with 95% confidence intervals. The size of each box indicated the weight of the study, while each crossed line refers to a 95% confidence interval with the mean effect at the center. The possibility of publication bias was assessed visually with funnel plots, and the objectivity test of Egger’s test with a *p*-value less than 0.05 was considered evidence of publication bias.

## 3. Results

### 3.1. Overview of Selected Studies

**Figure 1:**
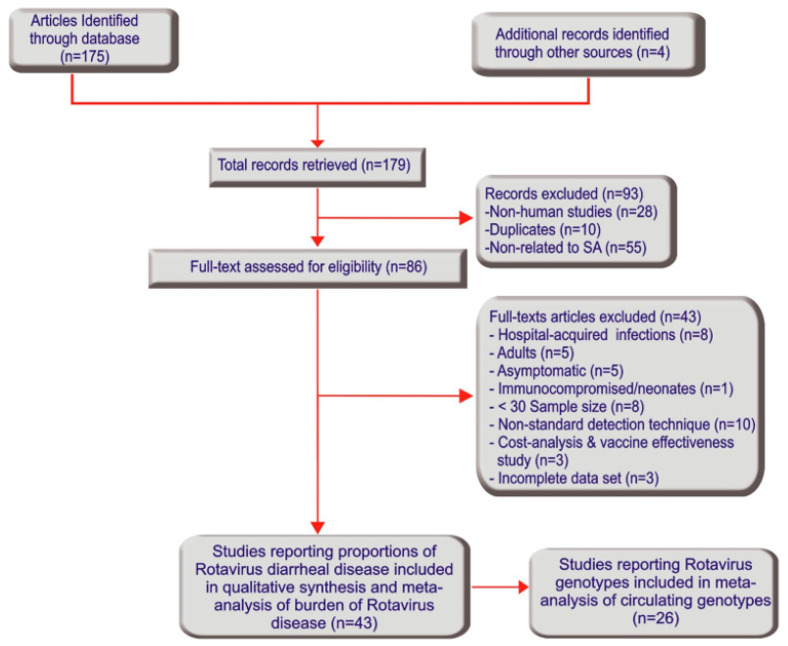
Study search and retrieval processes (Preferred Reporting Items for Systematic Reviews and Meta-Analyses flowchart).

A total of 179 research articles were identified through the electronic search and other sources. After reading through the titles and abstracts, 93 records were excluded due to duplication and lack of relevance. Thus, a total of 86 articles were screened and 41 full-texts were further excluded following full-text review. Forty-five full texts of the remaining 30 articles were further scrutinized and two full texts were excluded due to incomplete data sets. The remaining 28 articles, comprising 43 studies that satisfied the inclusion criteria and were of satisfactory quality, were included in the systematic study and meta-analysis of the burden of rotavirus disease (Figure 1 and Table 1). The individual study characteristics and quality assessment scores are depicted in Table 1. To obtain information on the geographical distribution of infection, studies reported from sentinel sites were placed in their respective province rather than by hospital name. The studies were conducted in all nine geo-political provinces of South Africa, namely, Gauteng (17), Eastern Cape (2), Free State (1), Limpopo (2), Mpumalanga (6), Northern Cape (1), North West (1), Western Cape (5), KwaZulu-Natal (8) (Table 1). Among the 43 studies, twenty-six studies reported rotavirus G and P genotypes typed by RT-PCR and were used for meta-analysis of circulating rotavirus genotypes (Figure 1 and Table 2).

### 3.2. Meta-Analysis of Prevalence of Rotavirus Infection among Children under Five in South Africa

The 43 studies detected rotavirus in 5659 samples out of 24,104 stool samples obtained from under-five children with acute gastroenteritis. The pooled prevalence using the random-effect model showed statistically significant heterogeneity between the studies. Hence, there was no need to perform analyses using the fixed-effects model. Thus, using the random-effects model, the estimated pooled prevalence of rotavirus infection among under-five children reported in the 43 studies was 24% (95% CI: 22%, 26%; I^2^ = 92.0%, *p* < 0.01).

#### 3.2.1. Meta-Analysis of the Estimate of Rotavirus Infection during the Pre-Vaccination Period

**Figure 2:**
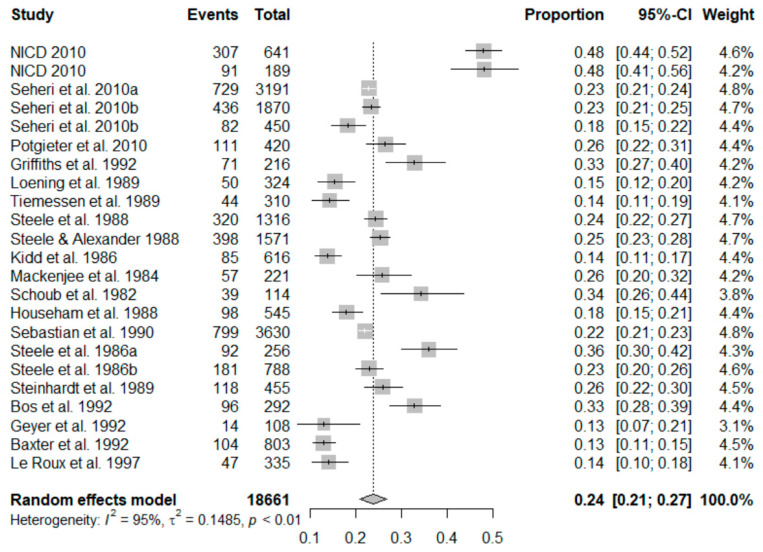
Forest plot showing the pooled prevalence of rotavirus cases before the inclusion of rotavirus vaccination in South Africa.

In the 23 studies that were included in the meta-analysis of pre-vaccination, the summary proportion estimated at 24% (95% CI: 21–27%) was obtainable as a random effect due to heterogeneity of estimates across studies. The I^2^ was 95.19% (95% CI: 95.26–98.66%) of the total variance between studies. Tau I^2^ was 15% (95% CI: 0.14–0.55%) (SE = 0.0681). The Q test statistic was Q (df = 22) = 457.2123, *p*-value < 0.0001), and it shows that the included studies did share a common effect size (Figure 2). The presence of publication bias was examined using funnel plots and tests (Egger’s and Begg). A visual inspection of the consequential funnel plot discovered asymmetrical distribution of the study findings. Nevertheless, the impartial assessment of bias using the Egger’s regression test was (z = −0.9253, *p* = 0.3548), which indicated that there was no evidence of publication bias. 

#### 3.2.2. Meta-Analysis of the Estimate of Rotavirus Infection during the Post-Vaccination Period

**Figure 3:**
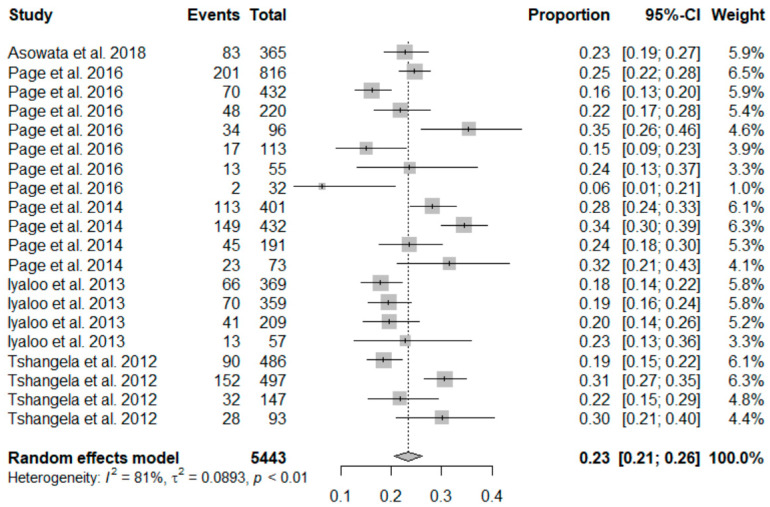
Forest plot showing the pooled prevalence of rotavirus cases after the inclusion of rotavirus vaccination in South Africa.

**Figure 4:**
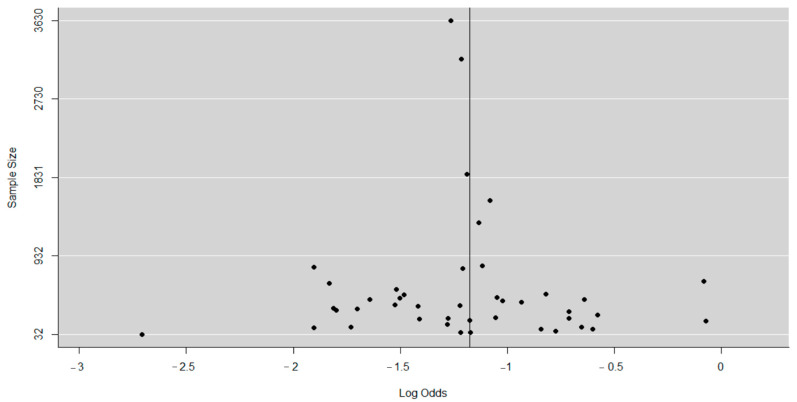
Funnel plot showing the presence of publication bias.

**Figure 5:**
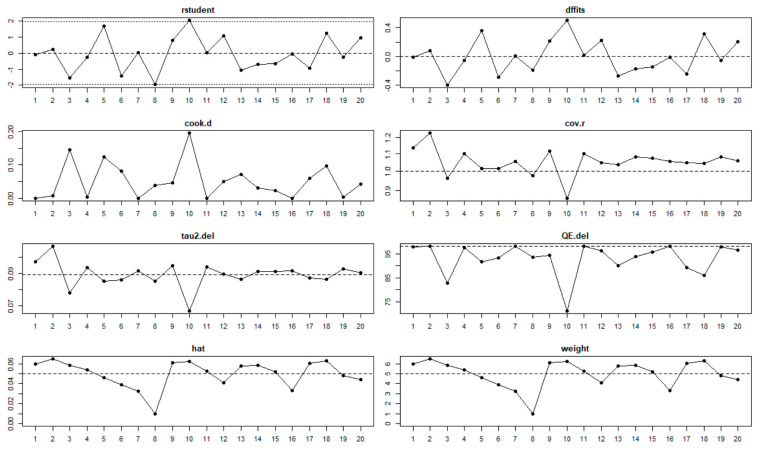
Baujat plot showing that no single study influences the results.

The findings for post-vaccination summary proportion using meta-analysis of random effect was 23% (95% CI: 21–25%). The heterogeneity denoted by obtained I^2^ was 80.67% (95% CI: 65.02–93.08%) as the total variance between studies, and the settings used in the article that met the inclusion criteria. Additionally, the estimated amount of total heterogeneity represented by Tau I^2^ was 9% (95% CI: 0.04–0.29%) (SE = 0.0421). The chi-square test statistic, Q (df = 19) = 98.3099, *p*-value < 0.0001) affirmed that the included studies shared a common effect size. Thus, we concluded that our analysis had substantial homogeneity (Figure 3). However, the Egger’s regression test was conducted, and it showed that there was no evidence of statistically significant publication bias (z = −1.3976, *p* = 0.1622). The visual assessment of the publication bias revealed an asymmetrical distribution for the overall data that met the inclusion criteria (Figure 4). Additionally, each point in the plot represents a separate study. The vertical axis represents the sample size; the horizontal axis represents the log odds of estimates and the asymmetric of the plot signalizes no publication bias. Finally, the diagnostic test to detect sources of heterogeneity in meta-analytic data (that is Baujat plots) was performed. This plot shows the involvement of each separate study to the overall Q-test statistic for heterogeneity on the horizontal axis against the influence of each separate study. Based on the findings from this study, the plot showed that there was no single study that influenced the results (Figure 5).

#### 3.2.3. Subgroup Analysis

**Figure 6:**
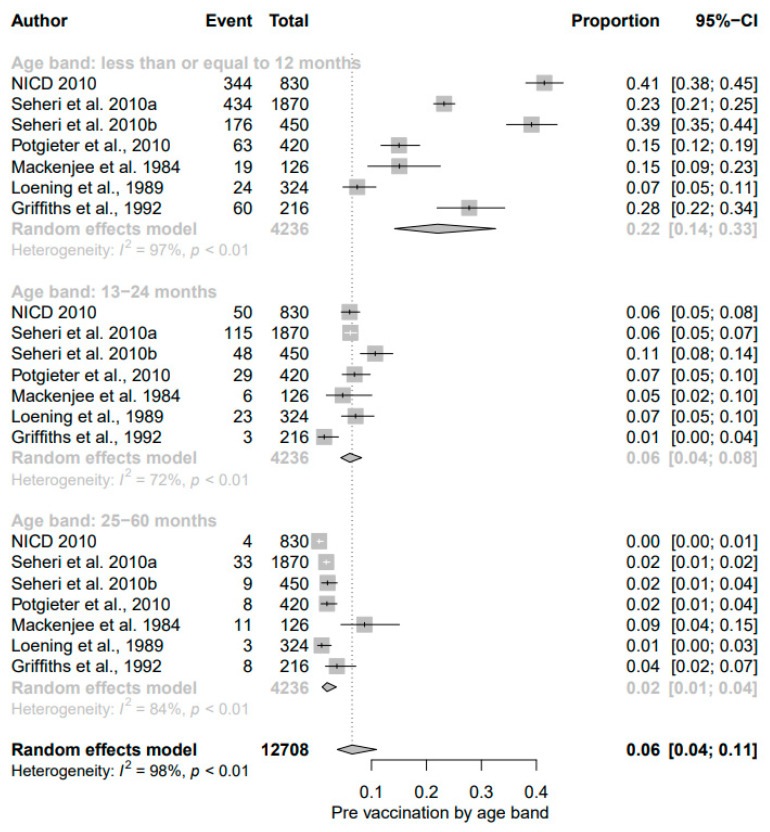
Subgroup analysis of rotavirus prevalence during pre-vaccination era according to age group.

**Figure 7:**
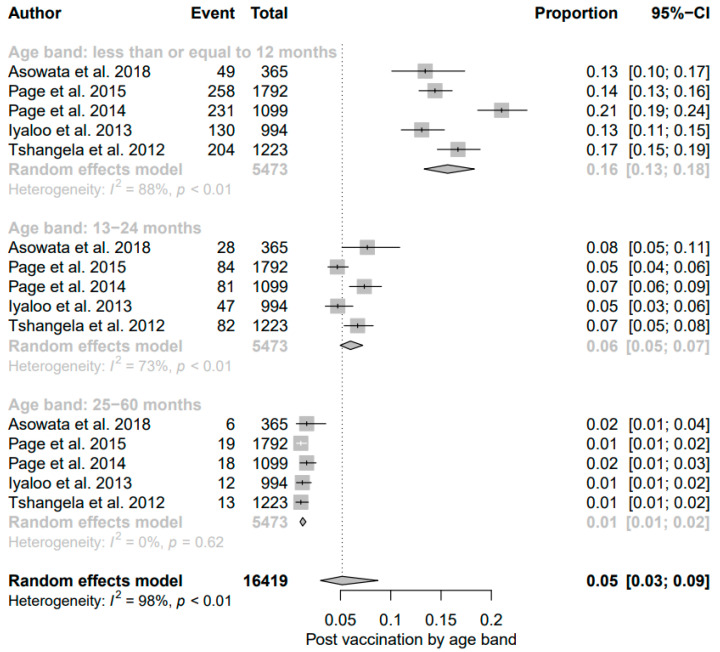
Subgroup analysis of rotavirus prevalence during post-vaccination era according to age group.

Subgroup analyses (Supplementary Figures S1–S3, Figure 6 and Figure 7) were carried out according to Province, study period, age, and study settings, respectively. In the analysis by Province pre- and post-vaccination (Supplementary Figures S1 and S2), the geographical distribution of rotavirus-attributable diarrhea in the pre-vaccine era indicated that studies conducted in Mpumalanga and Gauteng province accounted for the highest prevalence, at 28% and 25%, respectively, while a lower prevalence was found in studies conducted in KwaZulu Natal and Eastern Cape, with rates of 21% (95% CI: 17%, 26%) and 21% (95% CI: 10%, 38%), respectively. Following vaccine introduction, a significant decline in the prevalence of rotavirus infection was observed in Mpumalanga province from 28% (95% CI: 11, 57) in the pre-vaccine era to 22% (95% CI: 19, 25), while the Kwazulu-Natal region was noted to have a substantial rise from 21% (95% CI: 17%, 26%) in the pre-vaccine era to 28% (95% CI: 23%, 33%); I^2^ = 50.19% following vaccine licensure. However, a slight decline in rotavirus diarrhea was observed in Gauteng (25% to 22%). The rotavirus prevalence stratified according to Province showed high heterogeneity in both the pre- and post-vaccination eras, possibly due to variation in sample size, study design, or differences in characteristics of patients investigated.

The subgroup analysis by study period was conducted to assess the potential heterogeneity between studies carried out during the post-vaccination era. Of the 20 studies, the highest estimated prevalence was found in studies conducted in 2013 (12 months) (29% (95% CI: 25%, 34%), I^2^ = 65%) followed by studies conducted in 2011 (12 months) (25% (95% CI: 20% to 31%), I^2^ = 86%) while the lowest estimated prevalence was found in studies conducted in 2012 (12 months) (19% (95% CI: 17%, 22%)) (Supplementary Figure S3).

The result of subgroup analysis in relation to age groups showed a significant reduction in prevalence of diarrheal cases due to rotavirus among children aged ≤12 months following vaccine introduction; the detection rate (22% (95% CI: 14%, 33%); I^2^ = 97%) in the pre-vaccine era declined to 16% ((95% CI: 13%, 18%); I^2^ = 88%, *p* < 0.01) in the post-vaccine era (Figure 6 and Figure 7). While the overall prevalence remained unchanged between the two different periods for those aged 13–24 months, a decline of 1% was observed among older children in the post-vaccine era.

A subgroup analysis executed to assess the weight of rotavirus infection on diarrheal disease according to the settings of studies (outpatient department, hospital, or community-based) showed that both the hospital-admitted children and outpatient cases due to rotavirus diarrhea declined from 24% each during the pre-vaccination period to 23% each in the post-vaccination period, while the lowest proportion was found for community-based studies (Supplementary Figures S4 and S5).

### 3.3. Rotavirus Genotype Distribution in South Africa

Information on the circulating G- and P-type rotavirus strains was available for 3591 isolates from all 31 studies typed using RT-PCR, and is presented in Table 1. The genotype constellations observed were grouped according to the criteria used in Iturriza-Gómara [69] (Table 2). Overall, the most frequent circulating G type was G1 (37%), followed by G2 (22%), and G9 (10%), while genotypes G3, G12, G8, and G4, respectively, accounted for 9%, 7%, 4%, and 2% of the infections (Supplementary Figure S6). Similarly, P[8] (55%), P[4] (20%), and P[6] (15%) were the most prevalent P types nationally. Mixed genotypes and strains not typed accounted for 7% and 10% of the isolates, respectively (Supplementary Figure S7).

The predominant G genotypes circulating in South Africa before the introduction of the vaccine were G1 (48%), followed by G2 (19%), and G3 (12%). After vaccine introduction, G2 (27%) became the most dominant strains followed by G9 (25%), and G12 (15%) (Supplementary Figure S6). The predominant P strains in the pre-vaccination period were P[8] (54%), followed by P[6] (19%), and P[4] (16%). In the post-vaccination period, P[8] (52%) still predominates, followed by the rapidly evolving P[4] (30%) and the diminishing P[6] (7%) strains (Supplementary Figure S7).

The most common G/P genotype combinations identified in South Africa were: G1P[8] (32.21%), G2P[4] (16.85%), G9P[8] (9.02%), and G12P[8] (5.88%). Genotype combinations G1P[8] (43.13%), G2P[4] (14.55%), G3P[8] (7.02%), and G1P[6] (5.52%) were the leading G/P combinations before the use of the vaccine was implemented. Following vaccine implementation, G9P[8] (23.22%), G2P[4] (21.2%), G12P[8] (14.3) and G1P[8] (11.53%) became the dominant circulating strains within the country. A substantial decline of G1P[8] strains that predominated in the pre-vaccine era heralded the introduction of rotavirus vaccines in favor of the previously uncommon strains gaining more epidemiological relevance (Table 2).

**Table 2:** Circulating rotavirus genotype G/P combinations in South Africa grouped by analogy to those of Iturriza Gómara [69].

Genotypes	Post-Vaccination		Pre-Vaccination		*p*-Values	Total Genotypes	
	n	%	n	%		n	%
Common human rotavirus genotypes							
G1P[8]	143	11.53	1014	43.13	<0.0001	1157	32.21
G2P[4]	263	21.20	342	14.55	0.001	605	16.85
G3P[8]	8	0.65	165	7.02	<0.0001	173	4.82
G4P[8]	1	0.08	1	0.04	1.000	2	0.05
G9P[8]	288	23.22	36	1.53	<0.0001	324	9.02
Reassortment among common human rotavirus genotypes							
G1P[4]	8	0.65	12	0.51	0.371	20	0.56
G2P[8]	3	0.24	4	0.71	0.705	7	0.19
G3P[4]	1	0.08	1	0.09	0.564	3	0.08
Potential zoonotic rotavirus genotypes							
G3P[3]	43	3.47	0	0.00	-	43	1.19
G2P[6]	0	0.00	111	4.72	-	111	3.09
G8P[6]	0	0.00	15	0.65	-	15	0.42
G9P[6]	10	0.81	30	1.27	0.002	40	1.11
G9P[10]	1	0.08	0	0.00	-	1	0.02
Possible human-animal hybrid rotavirus genotypes							
G1P[6]	3	0.24	130	5.52	<0.0001	133	3.70
G2P[6]	66	5.32	108	4.59	0.001	174	4.85
G4P[6]	2	0.16	2	0.09	1.000	4	0.11
G8P[4]	84	6.77	30	1.28	<0.0001	114	3.17
G8P[8]	28	2.26	21	0.89	0.317	49	1.36
G12P[4]	6	0.48	3	0.13	0.317	9	0.25
G12P[6]	9	0.73	35	1.49	<0.0001	44	1.23
G12P[8]	174	14.03	37	1.57	<0.0001	211	5.88
Mixed	37	2.98	107	4.55	<0.0001	144	4.01
Untypable	53	4.27	141	5.99	<0.0001	194	5.40

Note: ‘%’ columns represent the proportion of circulating rotavirus genotypes while the ‘*p*-values’ indicate the levels of statistical significance based on Chi square test.

## 4. Discussion

This review evaluated the status of rotavirus infection and the impact of rotavirus (RV) vaccine introduction on the prevalence, distribution of RV genotypes in South Africa. It also confirmed the important roles the existing RV surveillance systems have played following the early and widespread use of the vaccines in South Africa, as it has provided the opportunities for a large number of studies that facilitated this post-licensure evaluation. The pooled rotavirus prevalence among under-five children in South African study sites obtained in this study was 24% (95% CI: 22%, 26%), very similar to the 23% and 24.3% pooled rotavirus prevalence rates recently reported in Ethiopia [70] and the Caribbean regions and Latin America [71], respectively. The current rate is slightly lower than the overall pooled estimate of rotavirus prevalence of 26.90% reported from a meta-analysis study of under-five children with acute gastroenteritis in 18 sub-Saharan African countries [5]. This disparity could be attributed to differences in the burden of disease across study settings, sensitivity of the diagnostic assays used during these two different periods, as well as choice and characteristics of study subjects.

Our meta-analysis findings indicate that significant reductions in the numbers of hospital-admitted and outpatient cases, as well as an overall decline in the proportion of diarrhea episodes due to rotavirus, occurred among under-five children in South Africa following the introduction of the rotavirus vaccine to the national childhood immunization programs, as previously corroborated [45,48]. These observations are consistent with meta-analysis findings in sub-Saharan Africa [72], Ireland [73], and the Caribbean countries [71], and also provide further evidence that rotavirus vaccinations are associated with a reduction in rotavirus-diarrhea morbidity, emergency visits, and hospitalizations. Contrary to the findings from the subgroup analysis of the study settings, the little change of the pooled RV prevalence pre- and post-RV vaccine introduction may be due to the contributions of community-based cases not represented in either the hospital or outpatient cases, or uneven routine childhood immunization coverage among the Provinces, which has been consistently reported [74,75,76]. During 2016/2017, for example, a wide provincial variation in vaccine coverage was observed in which some regions like Limpopo experienced coverage as low as 64.5% [76]. The continuous struggle to attain the national immunization target and the heterogeneous nature of coverage may cause regional fluctuations which could impact on the overall diarrheic burden. Although there was a decline in the diarrheic burden post-vaccine inclusion, a pre-specified subgroup analysis revealed a rise in rotavirus diarrheal cases during the epidemiological year in 2013 after a biennial reduction similar to the trends in national rotavirus activity after the introduction of rotavirus vaccine into the national immunization program in the United States [77]. This fluctuation may be a result of reduced vaccine effectiveness in the preceding year in South Africa, which was estimated at only 57% after two doses [78]. The low rotavirus vaccine coverage experienced in 2013 as officially noted by the South African National Department of Health (estimated at 64%) by WHO and UNICEF [79], as well as possible changes in circulating genotypes could also have contributed to the observed peak of RV-associated diarrheal cases in the vaccine era. Despite the fluctuation in the disease trends in some epidemiological years, the decline of overall diarrheic burden in South Africa could influence rates of secondary healthcare use associated with rotavirus-attributable diarrheal cases in the post-vaccination period.

In South Africa, the observed diarrheal cases due to rotavirus varied considerably among age groups. Higher rotavirus prevalence was observed in infants, followed by those in the second year of life, while older ages had lower prevalence. This meta-analysis highlights the occurrence of a significant reduction of acute rotavirus gastroenteritis in infants among whom the highest burden of disease exists before rotavirus vaccination was executed in South Africa. This reduction could be due, in part, to the impact of the rotavirus vaccine, in which the introduction has made it easier to notice the dramatic decline in the diarrheic burden that was present in the population, but not noticeable, in the absence of vaccination. Our observation is consistent with the higher reduction observed in infants compared to older age groups following rotavirus vaccination in middle-income countries such as Brazil, Colombia, Nicaragua, and Bolivia [80], as well as other African countries [81]. The lack of sustained reduction in rotavirus prevalence noted among children in the second year of life, possibly due to waning immunity, has been observed previously in Malawi [82], Rwanda [83], Burkina Faso [84] and Ghana [85]. Nevertheless, increasing and sustaining high vaccine coverage, especially in provinces where a significant burden has been identified in the current era, may indirectly protect older children from re-infection through herd immunity, as indicated for Europe [86].

In this review, the globally common G (G1–G4, G9, G12) and P (P[4], P[6], and P[8]) rotavirus genotypes were also observed, although G3, G4, and G12 were reported at low prevalence. A change in genotypic predominance was observed following the introduction of the rotavirus vaccine into the national childhood immunization program in South Africa. The predominance of the G1 genotype during the pre-vaccine era and its decline (from 48% to 12%) and the emergence of G2, G9, and G12 during the post-vaccine era is similar to the trends recently reported in Australia [87] and Zambia [88]. This change could be a result of selective immunologic pressure of the vaccine on G1 or differential viral fitness among immunologically protected hosts. The implication of diverse emerging dominant strains in circulation coupled with the pool of viral reservoirs is that targeted efforts, such as the human vaccine and other intervention strategies, may become less effective for achieving complete elimination of the virus from the human population. The predominance of G2 strain in the post-vaccination era might be indicative of weaker vaccine protection against this genotype, which has been reported [89]. Therefore, a continuous monitoring of the presence of this genotype, including G9 and G12 strains, is imperative for ascertaining whether the increase in these heterotrophic genotypes, which are seemingly evading vaccine immunity, is a result of vaccine pressure or genotype evolution. An unusual genotype G8 virus of bovine origin capable of rapid adaptation to human populations was observed in 4% of South African children, probably pointing to the existence of dynamic interaction and interspecies transmission events between human and bovine rotaviruses, which could provide a mechanism for the generation of more genetic diversity through reassortment of genomes.

This meta-analysis indicates that the distribution of P-type genotypes is geographically less diverse than the G-types circulating in South Africa. While rotavirus of different G types predominates in both the pre-vaccine and post-vaccine periods, only the P[8] VP4 strain was found to predominate in both periods alongside other less dominant P[4] and P[6] genotypes. A recent meta-analysis reported by Damtie et al. [70] also identified a similar trend in P[8] predominance following vaccine introduction in Ethiopia. The findings of persistent P[8] dominance may be indicative of less vaccine protection against this genotype. Contrary to our findings, Carvalho-Costa et al. [90] reported genotype P[4] dominance a decade after the introduction of universal vaccination with Rotarix in Brazil, which was attributed to the prolonged effect of vaccine pressure on the P[8] or normal genotype fluctuations. With the rapidly increasing rate of P[4] (from 16% in the pre-vaccine era to 30% in the post-vaccine era) in South Africa, there is the likelihood of it overriding P[8] in subsequent years, as predicted by a mathematical model for countries where the Rotarix vaccine is used [91].

Several studies have documented the emergence and sudden increase in the proportion of rotavirus genotypes not represented in the vaccine formulation since the introduction of rotavirus vaccines into the national immunization programs [92,93]. In our review, a significant decline of G1P[8] (43.13% to 11.53%) and the emerging dominance of G9P[8] (23.22%), G2P[4] (21.2%), G12P[8] (14.3) in the vaccine era suggest that the introduction of the Rotarix vaccine can impose selective pressure on circulating strains, which could favor the shift toward otherwise less-dominant strains or the selection of mutant strains that were not adequately neutralized. A recent meta-analysis from Ethiopia and Europe also noted an increasing trend of G2P[4], G9P[8], and/or G12P[8] and other previously uncommon genotypes not fully represented in the monovalent Rotarix vaccine that covers the G1P[8] strain [70,94]. Similarly, a significant proportion of diarrheal episodes were consistently noted in an association with the heterotypic G2P[4] rotavirus genotype in Latin America, Belgium, Botswana, and Australia [87,95,96,97] and G9P[8] in northern Vietnam [98], despite reports of large-scale vaccination with the Rotarix vaccine. While some authors have attributed changing aspects of genotype distribution to lack of sufficient protections against heterologous and the newly emerging rotavirus strains, creating opportunities for strain selection due to vaccine-induced immunological pressures [87,99], others are of the opinion that natural strain fluctuation or gene reassortment events would be more likely to influence the emergence and the epidemiological fitness of variants in the absence of limited herd immunity [22,100]. Although the rotavirus vaccine has been shown to offer both homotypic and heterotypic immunity [101], a reduction in the level of vaccine protection against the emerging dominant non-G1 strains (G2, G9, and G12) circulating among South African children is a possibility, as low vaccine effectiveness of 62% against strains with the G or P in the vaccine formulation and 52% against strains without a G or P in the vaccine formulation has been previously observed in South Africa [78]. A meta-analysis report by Leshem et al. [94] did show evidence of low vaccine effectiveness against the heterologous strain in Latin America and Europe. Contrary to the significant decline of vaccine virus genotype and the increased frequency of detection of previously uncommon genotypes in South Africa, the human G1P[8] constellation remains predominant in countries such as Central African Republic and Benin where RV vaccine has not been fully established [25,102]. Additionally, the post-vaccination era also exhibited a significant increase of G2P[6] and G8P[4] species, with mosaic genotype constellation of human–animal origin, which were reported to be low in Benin [102]. The increase in rotavirus cases observed in the KwaZulu-Natal and Western Cape provinces despite the overall decline of diarrheal morbidity in the post-vaccine era may be explained, in part, by the accumulation of more strains that are heterotypic to vaccine types in a population, which was attributed to a higher disease burden in a meta-analysis from middle-income countries (Brazil, Colombia, Nicaragua, and Bolivia) [80]. However, the few pre-vaccine studies from the Western Cape province makes its comparison with several other post-vaccination studies from the region difficult. In general, the emerging dominance of non-vaccine genotypes combination (G9P[8], G2P[4], and G12P[8]) not fully represented in vaccine formulations have raised concerns regarding potential genotype replacement in disease, which may dampen the overall public health benefit of the vaccine. Therefore, the call for ongoing monitoring of disease trends alongside genotype distribution is key to identifying any potential effects associated with the dynamics of genotype changes in South Africa.

The limitations of studies included in this review include reporting bias and the lack of standard protocol for studies from local settings. Additionally, the use of different primer sets for RT-PCR could lead to differing results with regard to the reported circulating G/P genotypes. Nevertheless, the results of this review show that rotavirus-attributable diarrhea has declined in South Africa since the introduction of the vaccine.

In conclusion, this review provided evidence of a reduction in the national burden of rotavirus-associated diarrheal morbidity among under-five children following the introduction of the rotavirus vaccine into primary immunization programs in South Africa. However, a further look at the change in the geographical distribution of RV infection revealed a significant increase in diarrheal cases in the KwaZulu-Natal and Western Cape Provinces where a decreasing trend in vaccine coverage has been documented. The vaccine licensure era presented a greater genotype diversity, including the emergence of the unusual G8 and G12 rotavirus strains commonly detected in animals. The dynamics of strain predominance between the two periods are either a function of vaccine-induced selective pressure or normal genotype fluctuations. Clear evidence of the trigger will require continued surveillance for rotavirus strain diversity and close monitoring of the long-term effects of vaccination on the genetic variants especially in Provinces where the diarrheic burden is still significant. This notwithstanding, the pooled and up-to-date epidemiological information from this review will guide policy-making processes for long-term use of the vaccine and the evidence of vaccine impact could serve to boost vaccine coverage generally.

## Figures and Tables

**Table 1:** Descriptive characteristics of included studies in South Africa.

Author	Year of Publication	Vaccination era	Province	Study Setting	Design	Duration /Period	Sample size	Assay Method	Age Band	No. (%) of Rotavirus Positive Cases	“Quality Score (A = 9–12) (B = 5–8) (C = 1–4)”	Reference
Asowata et al.	2018	Post-vaccine	Kwazulu-Natal	Outpatients	Cross-sectional	2014–2015	365	ELISA, RT-PCR	<5 years	83 (23)	B	[11]
Page et al.	2016	Post-vaccine	Gauteng	Hospitalized	Sentinel surveillance	2014–2015	816	IDEIA, RT-PCR	<5 years	201 (24.6)	A	[45]
Page et al.	2016	Post-vaccine	Western Cape	Hospitalized	Sentinel surveillance	2014–2015	432	IDEIA, RT-PCR	<5 years	70 (16.2)	A	[45]
Page et al.	2016	Post-vaccine	Mpumalanga	Hospitalized	Sentinel surveillance	2014–2015	220	IDEIA, RT-PCR	<5 years	48 (21.8)	A	[45]
Page et al.	2016	Post-vaccine	Kwazulu-Natal	Hospitalized	Sentinel surveillance	2014–2015	96	IDEIA, RT-PCR	<5 years	34 (35.4)	A	[45]
Page et al.	2016	Post-vaccine	Free state	Hospitalized	Sentinel surveillance	April–Dec., 2015	113	IDEIA, RT-PCR	<5 years	17 (15.0)	A	[45]
Page et al.	2016	Post-vaccine	Northern Cape	Hospitalized	Sentinel surveillance	April–Dec., 2015	55	IDEIA, RT-PCR	<5 years	13 (23.6)	B	[45]
Page et al.	2016	Post-vaccine	Limpopo	Hospitalized	Sentinel surveillance	April–Dec., 2015	32	IDEIA, RT-PCR	<5 years	2 (6.3)	B	[45]
Page et al.	2014	Post-vaccine	Gauteng	Hospitalized	Sentinel surveillance	2013 (12 months)	401	ELISA, RT-PCR	<5 years	113 (28.2)	A	[46]
Page et al.	2014	Post-vaccine	Western Cape	Hospitalized	Sentinel surveillance	2013 (12 months)	432	ELISA, RT-PCR	<5 years	149 (34.3)	A	[46]
Page et al.	2014	Post-vaccine	Mpumalanga	Hospitalized	Sentinel surveillance	2013 (12 months)	191	ELISA, RT-PCR	<5 years	45 (23.6)	A	[46]
Page et al.	2014	Post-vaccine	Kwazulu-Natal	Hospitalized	Sentinel surveillance	2013 (12 months)	73	ELISA, RT-PCR	<5 years	23 (31.5)	B	[46]
Iyaloo et al.	2013	Post-vaccine	Gauteng	Hospitalized	Sentinel surveillance	2012 (12 months)	369	ELISA, RT-PCR	<5 years	66 (17.9)	A	[35]
Iyaloo et al.	2013	Post-vaccine	Western Cape	Hospitalized	Sentinel surveillance	2012 (12 months)	359	ELISA, RT-PCR	<5 years	70 (19.5)	A	[35]
Iyaloo et al.	2013	Post-vaccine	Mpumalanga	Hospitalized	Sentinel surveillance	2012 (12 months)	209	ELISA, RT-PCR	<5 years	41 (19.6)	A	[35]
Iyaloo et al.	2013	Post-vaccine	Kwazulu-Natal	Hospitalized	Sentinel surveillance	2012 (12 months)	57	ELISA, RT-PCR	<5 years	13 (22.8)	B	[35]
Tshangela et al.	2012	Post-vaccine	Gauteng	Hospitalized	Sentinel surveillance	2011 (12 months)	486	ELISA, RT-PCR	<5 years	90 (18.5)	A	[47]
Tshangela et al.	2012	Post-vaccine	Western Cape	Hospitalized	Sentinel surveillance	2011 (12 months)	497	ELISA, RT-PCR	<5 years	152 (30.6)	A	[47]
Tshangela et al.	2012	Post-vaccine	Mpumalanga	Hospitalized	Sentinel surveillance	2011 (12 months)	147	ELISA, RT-PCR	<5 years	32 (21.8)	A	[47]
Tshangela et al.	2012	Post-vaccine	Kwazulu-Natal	Hospitalized	Sentinel surveillance	2011 (12 months)	93	ELISA, RT-PCR	<5 years	28 (30.1)	B	[47]
NICD	2010	Pre-vaccine	Gauteng	Hospitalized	Sentinel surveillance	2009 (7 months)	641	ELISA, RT-PCR	<5 years	307 (47.9)	A	[48]
NICD	2010	Pre-vaccine	Mpumalanga	Hospitalized	Sentinel surveillance	2009 (7 months)	189	ELISA, RT-PCR	<5 years	91 (48.1)	A	[48]
Seheri et al.	2010a	Pre-vaccine	Gauteng	Hospitalized	Sentinel surveillance	2003–2006	3191	IDEIA, RT-PCR	<5 years	729 (22.8)	A	[49]
Seheri et al.	2010b	Pre-vaccine	Gauteng	Hospitalized	Sentinel surveillance	2003–2005	1870	IDEIA RT-PCR	<5 years	436 (23.3)	A	[50]
Seheri et al.	2010b	Pre-vaccine	North West	Hospitalized	Sentinel surveillance	2004–2005	450	IDEIA RT-PCR	<5 years	82 (18.2)	A	[50]
Potgieter et al.	2010	Pre-vaccine	Limpopo	Outpatients	Cross-sectional	1998–2000	420	ELISA, PAGE, RT-PCR	<5 years	111 (26.4)	A	[51]
Le Roux et al.	1997	Pre-vaccine	Gauteng	Hospitalized	Cross-sectional	1996–1997	335	ELISA	<2 years	47 (14)	B	[52]
Bos et al.	1992	Pre-vaccine	Gauteng	Hospitalized	Cross-sectional	1989 (12 months)	292	ELISA	<3 years	96 (33)	A	[53]
Geyer et al.	1992	Pre-vaccine	Gauteng	Hospitalized	Cross-sectional	1988 (6 months)	108	ELISA	<3 years	14 (13)	B	[54]
Baxter et al.	1992	Pre-vaccine	Eastern Cape	Hospitalized	Cross-sectional	1989–1990	803	ELISA	<2 years	104 (13)	A	[55]
Griffiths et al.	1992	Pre-vaccine	Eastern Cape	Outpatients	Cross-sectional	1988–1989	216	IDEIA, PAGE, EM	<5 years	71 (32.9)	A	[56]
Sebastian	1990	Pre-vaccine	Kwazulu-Natal	Hospitalized	Cross-sectional	1984–1985	3630	ELISA	<2 years	799 (22)	B	[57]
Loening et al.	1989	Pre-vaccine	Kwazulu-Natal	Community-based	Cross-sectional	1985–1986	324	ELISA	<5 years	50 (15.4)	A	[58]
Tiemessen et al.	1989	Pre-vaccine	Mpumalanga	Outpatients	Cross-sectional	1985–1986	310	ELISA, EM	<2 years	44 (14.2)	A	[59]
Steinhardt et al.	1989	Pre-vaccine	Gauteng	Hospitalized	Cross-sectional	1984–1985	455	EM	<4 years	118 (26)	C	[60]
Steele et al.,	1988	Pre-vaccine	Gauteng	Hospitalized	Cross-sectional	1983–1986	1316	ELISA, EM	<5 years	320 (24.3)	A	[61]
Steele and Alexander	1988	Pre-vaccine	Gauteng	Hospitalized	Cross-sectional	1983–1986	1571	ELISA	<5 years	398 (25)	A	[62]
Househam et al.	1988	Pre-vaccine	Western Cape	Hospitalized	Cross-sectional	1981–1982	545	ELISA	<2 years	98 (18)	B	[63]
Steele et al.	1986a	Pre-vaccine	Gauteng	Hospitalized	Cross-sectional	1982 (10 months)	256	ELISA	<3 years	92 (36.0)	B	[64]
Steele et al.,	1986b	Pre-vaccine	Gauteng	Hospitalized	Cross-sectional	1983–1985	788	ELISA	<3 years	181 (23)	A	[65]
Kidd et al.	1986	Pre-vaccine	Gauteng	Hospitalized	Cross-sectional	1982–1983	616	ELISA	<2 years	85 (13.8)	B	[66]
Mackenjee et al.	1984	Pre-vaccine	Kwazulu-Natal	Outpatients	Cross-sectional	1982–1983	221	ELISA	<2 years	57 (25.8)	B	[67]
Schoub et al.	1982	Pre-vaccine	Gauteng	Hospitalized	Cross-sectional	1981 (1 year)	114	ELISA, EM	<2 years	39 (34.2)	B	[68]

EIA = enzyme immune assay, RT-PCR = reverse transcriptase–polymerase chain reaction, EM = electron microscopy.

## Supplementary Materials

The following are available online at https://www.mdpi.com/article/10.3390/v13101905/s1, Figure S1: Forest plot showing the pooled prevalence of rotavirus cases by Province before the inclusion of rotavirus vaccination in South Africa, Figure S2: Forest plot showing the pooled prevalence of rotavirus cases by Province after the inclusion of rotavirus vaccination in South Africa, Figure S3: Subgroup analysis of rotavirus prevalence post-vaccination era according to study periods, Figure S4: Subgroup analysis of rotavirus prevalence during pre-vaccination according to settings (outpatients vs. hospital vs. community), Figure S5: Subgroup analysis of rotavirus prevalence during post-vaccination according to settings (outpatients vs. hospital), Figure S6: Rotavirus G genotype distribution pre- and post-vaccine introduction in South Africa (1982–2020), Figure S7: Rotavirus P genotype distribution pre- and post-vaccine introduction in South Africa (1982–2020).
