# Supplementary material for: Prevalence, Pattern and Genetic Diversity of Rotaviruses among Children under 5 Years of Age with Acute Gastroenteritis in South Africa: A Systematic Review and Meta-Analysis
Source: Viruses. 2021 Sep 23;13(10):1905. doi: 10.3390/v13101905 (PMC8538439; doi:10.3390/v13101905)
Supplement: Supplementary file 1 [file viruses-13-01905-s001.zip › viruses-1351771-supplementary.pdf]

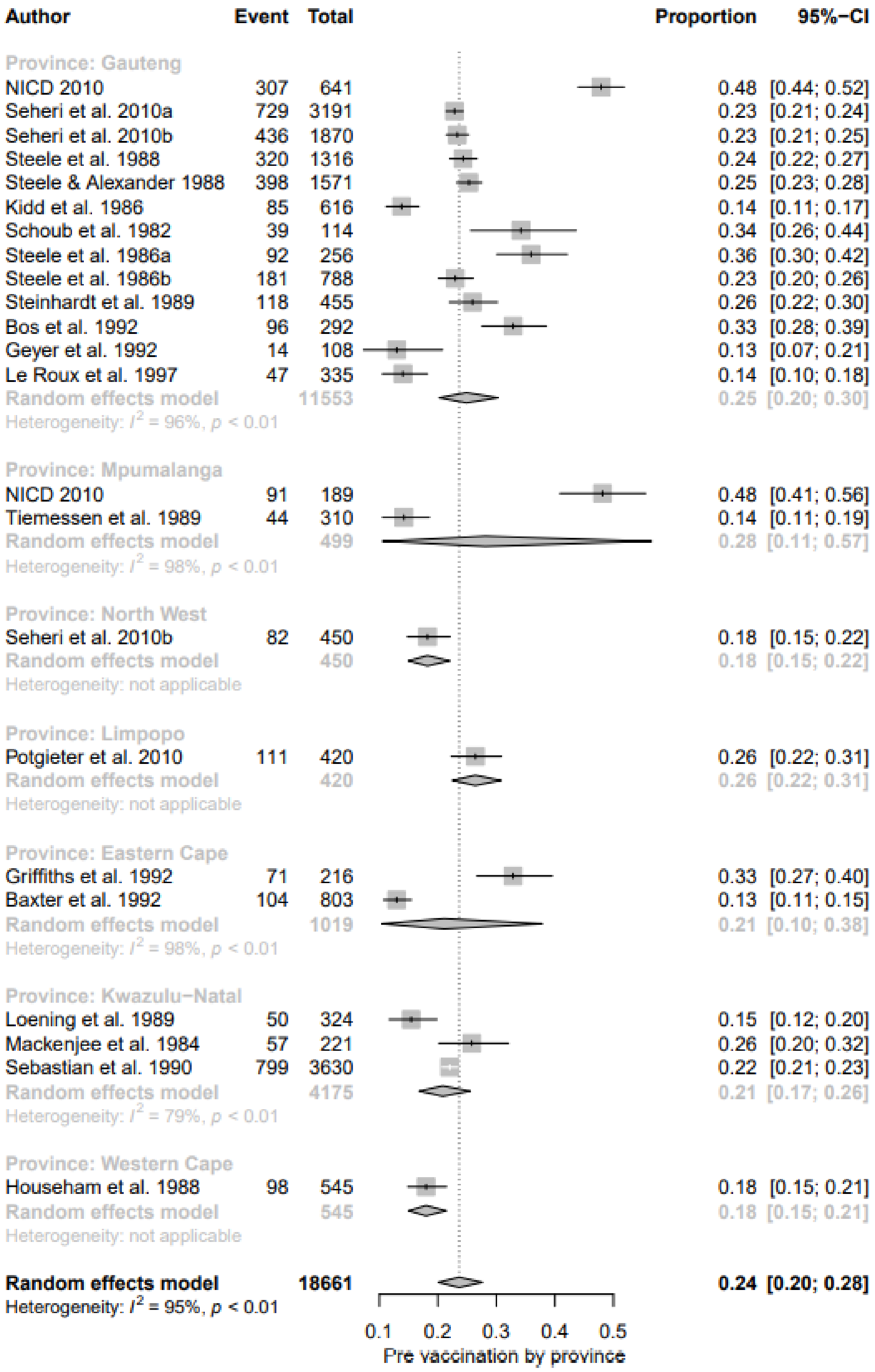

Figure S1. Forest plot showing the pooled prevalence of rotavirus cases before the inclusion of rotavirus vaccination in South Africa.

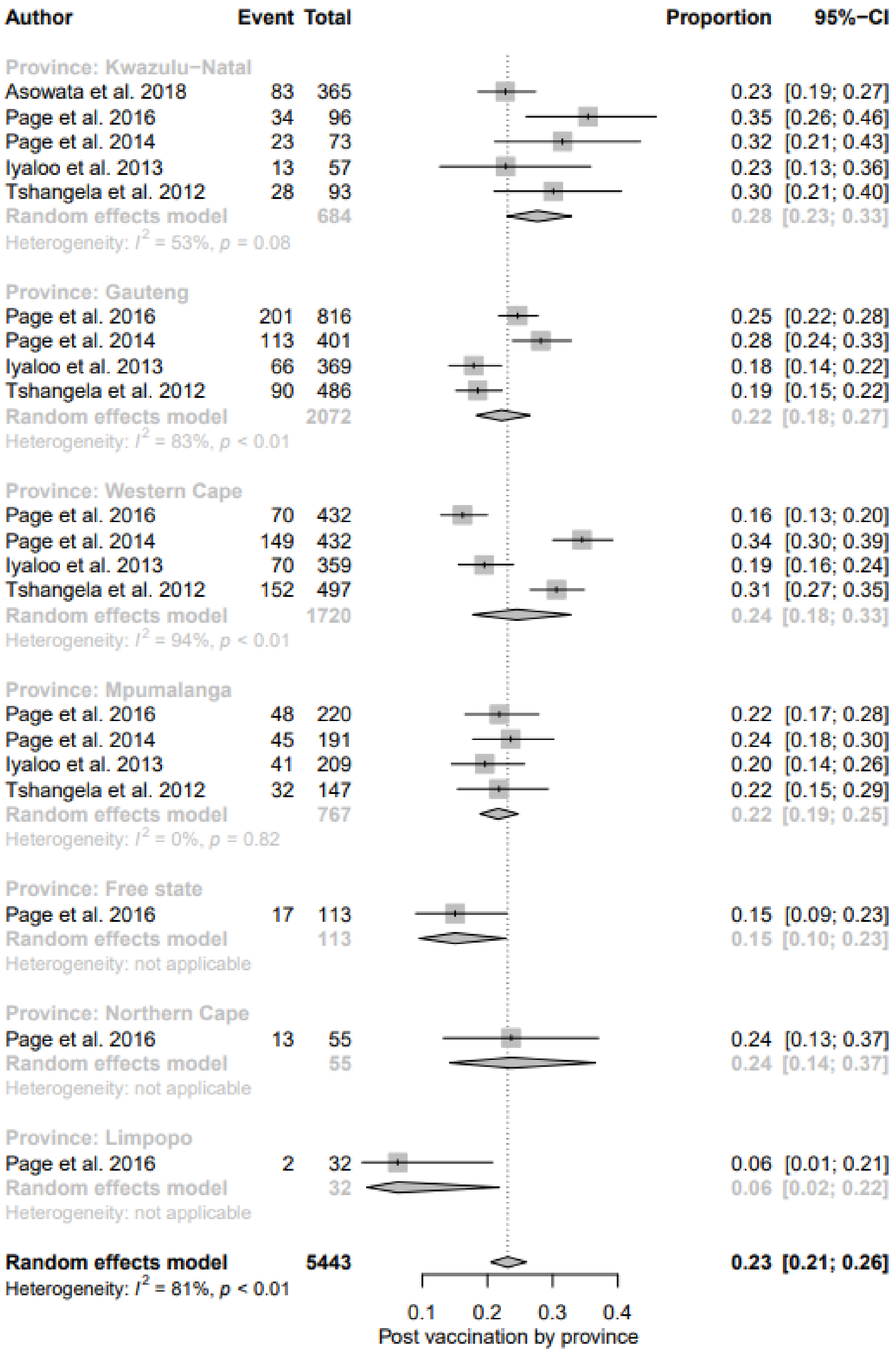

Figure S2. Forest plot showing the pooled prevalence of rotavirus cases after the inclusion of rotavirus vaccination in South Africa.

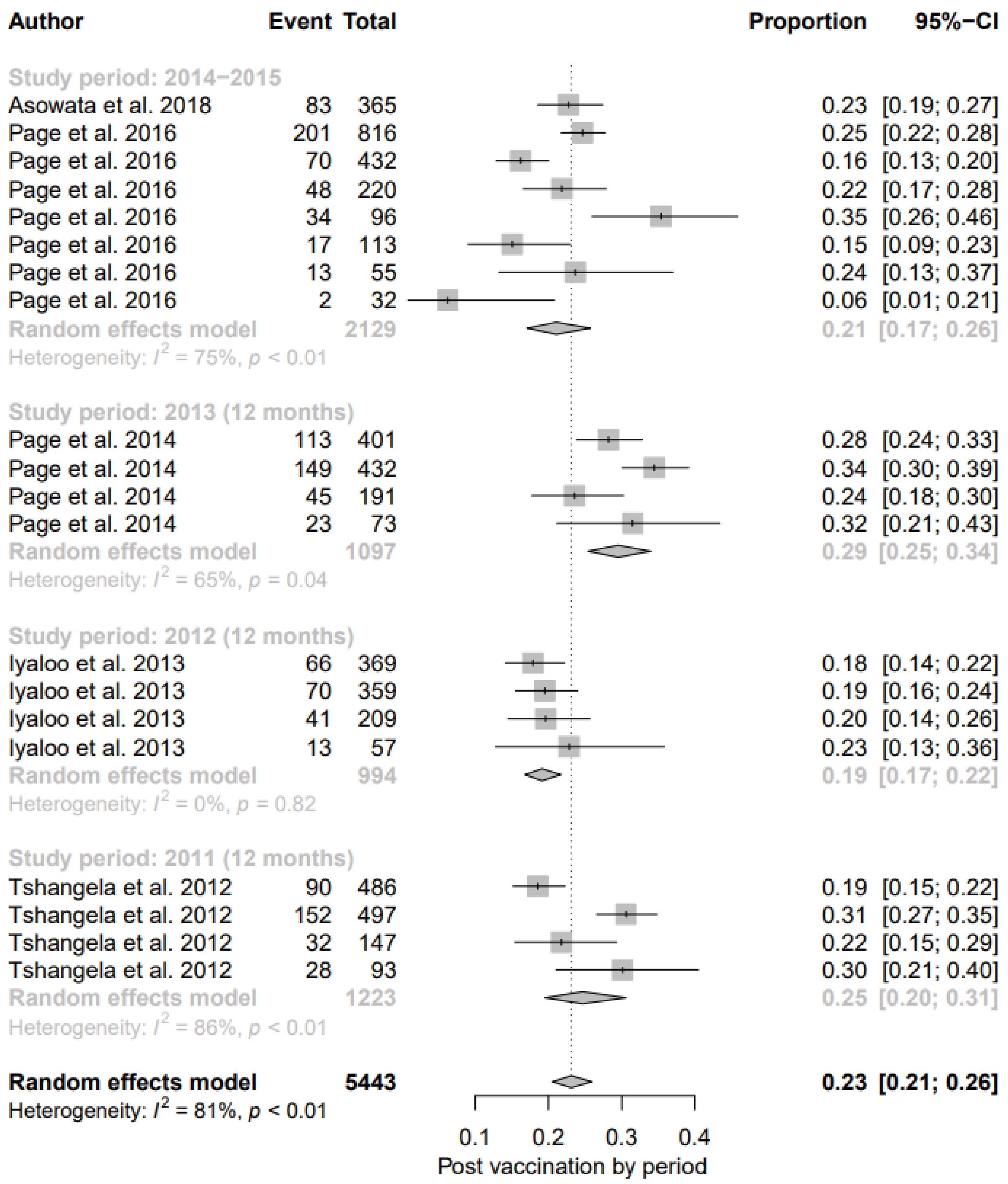

Figure S3. Subgroup analysis of rotavirus prevalence post-vaccination era according to study periods.

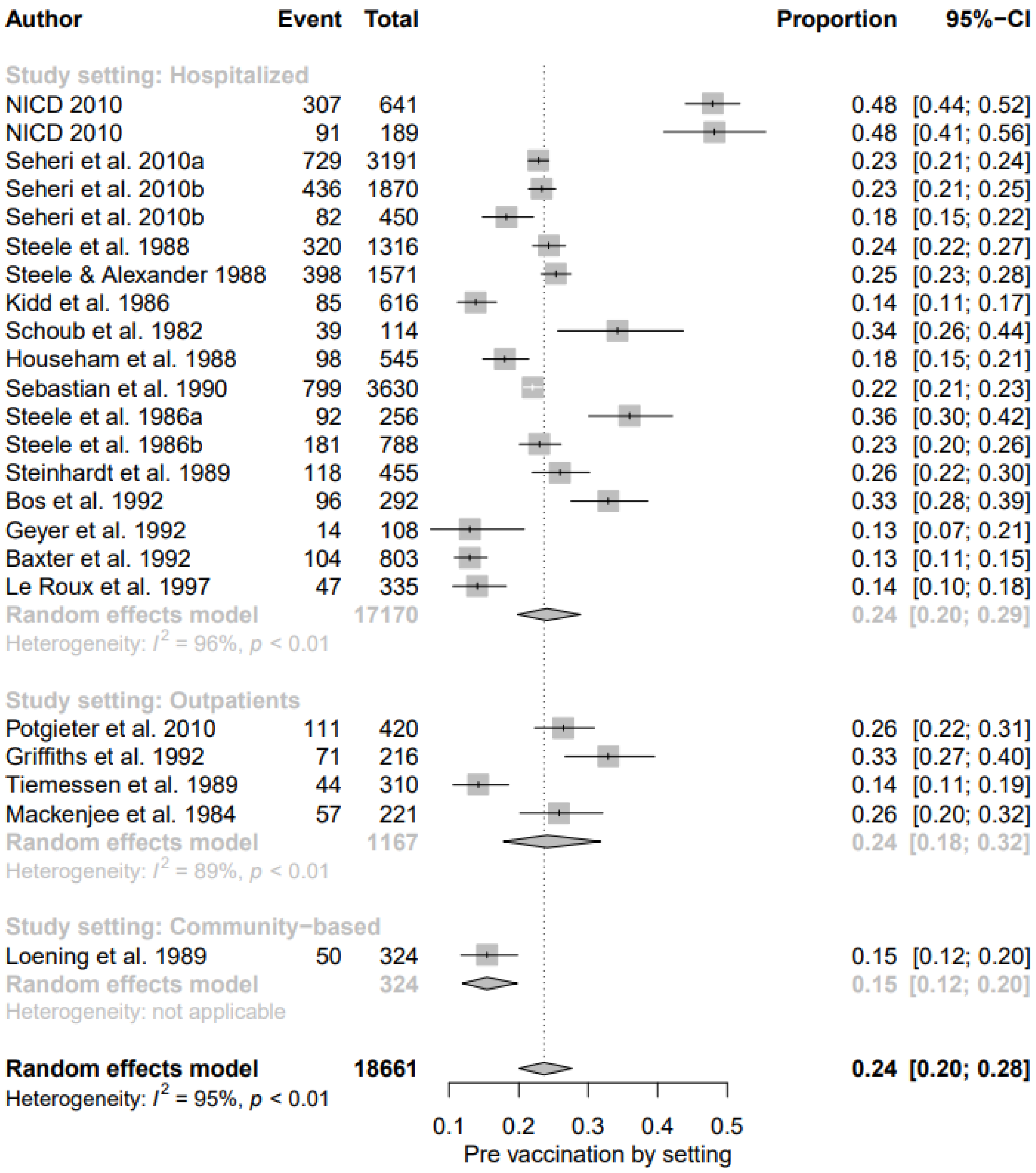

Figure S4. Subgroup analysis of rotavirus prevalence during pre-vaccination according to settings (outpatients vs. hospital vs. community).

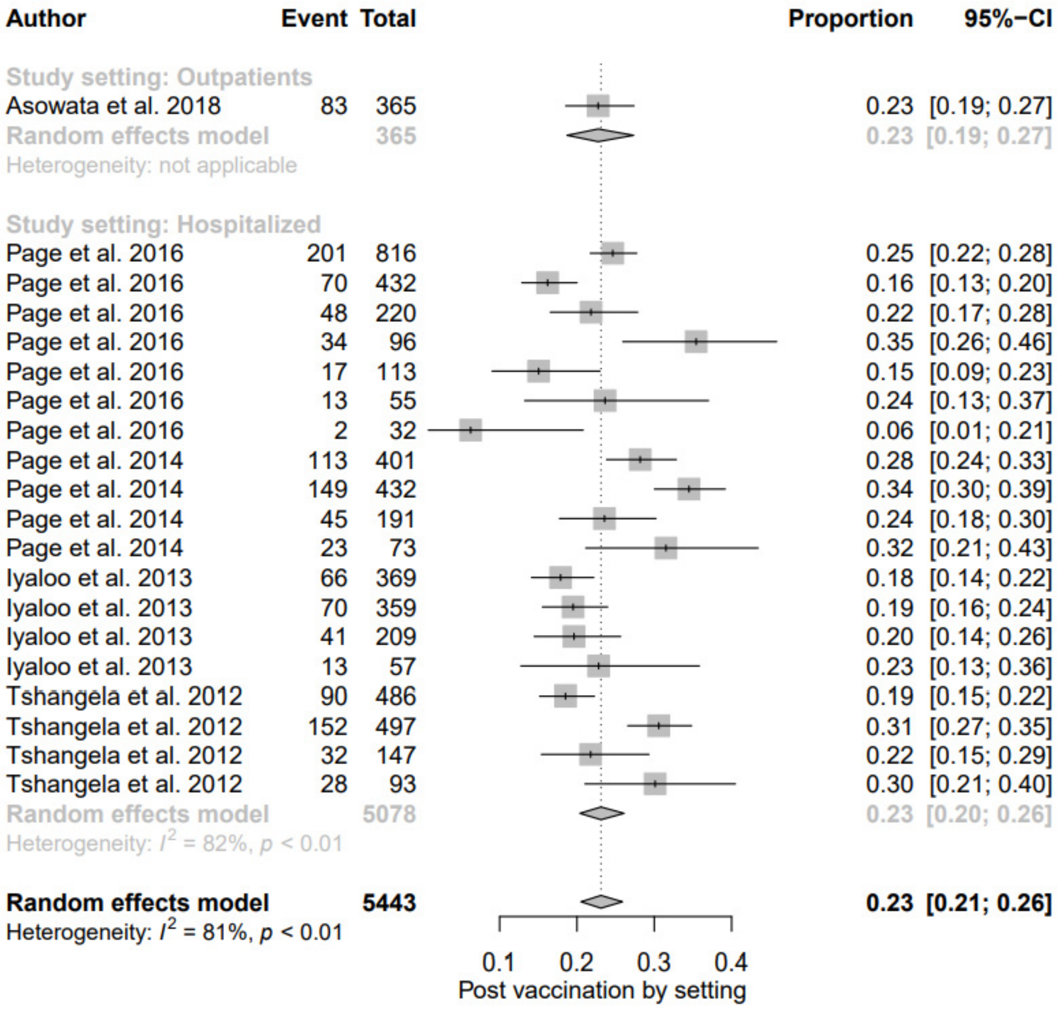

Figure S5. Subgroup analysis of rotavirus prevalence during post-vaccination according to settings (outpatients vs. hospital).

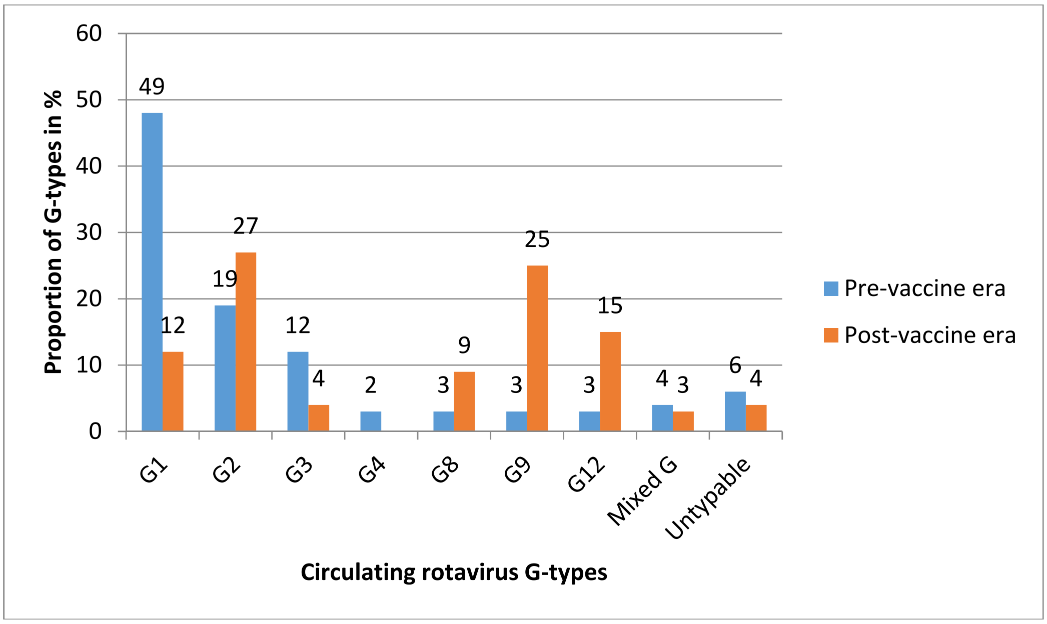

Figure S6. Rotavirus G genotype distribution pre- and post-vaccine introduction in South Africa (1982-2020).

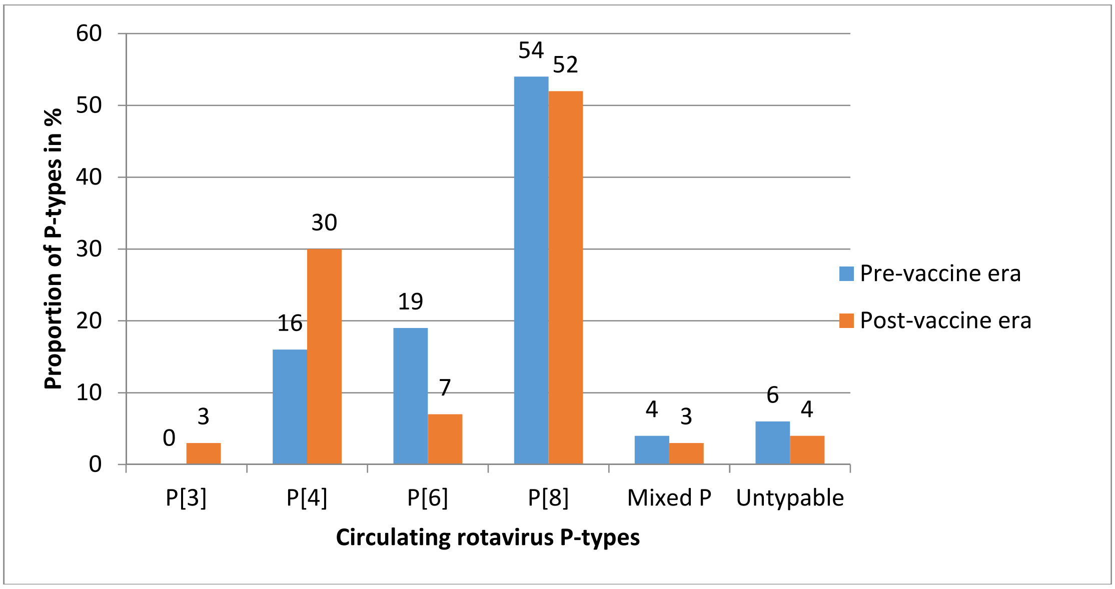

Figure S7. Rotavirus P genotype distribution pre- and post-vaccine introduction in South Africa (1982-2020).
